# Disrupted Intercellular Communication Causes a Disfiguring Birth Defect

**DOI:** 10.1371/journal.pbio.0040335

**Published:** 2006-09-12

**Authors:** Liza Gross

Before a fertilized egg begins the repeated rounds of cell division that turn the single cell into a proliferating, streaming, differentiating mass of cells, its fate may already be sealed. Inherited mutations in genes involved in segregating and sorting embryonic cells can result in serious abnormalities in body patterning and appear to underlie an inherited X-linked disorder (so-called because the mutated genes lie on the X chromosome) called craniofrontonasal syndrome (CFNS). X-linked disorders tend to affect males more severely than females, because boys inherit just one X chromosome while girls inherit two: if one gene is defective, the other can fill in. CFNS is a rare departure from this pattern, with females exhibiting the most severe symptoms. This disfiguring disorder is characterized by a range of skull aberrations, including facial asymmetry, widely spaced eyes, and abnormal head shape, as well as polydactyly and fused digits.

A class of receptor protein-tyrosine kinases called Ephs and their ephrin binding partners (called ligands) regulate tissue patterning by restricting cell interactions, ensuring proper cell sorting, and establishing developmental compartment boundaries. Mutations in one ephrin gene, *ephrin-B1*, have been identified in patients with CFNS and have been associated with aberrant skeletal patterning in mutant “heterozygous” female mice, which carry one normal and one nonfunctional copy of the *ephrin-B1* gene. Mutations in connexins, structural proteins that form gap junction pores, also lead to cranial and skeletal defects in both mice and humans.

In a new study, Alice Davy, Jeffrey Bush, and Philippe Soriano elucidate the mechanisms of ephrin-mediated cell sorting, and show how the breakdown of the process causes physical abnormalities. The researchers worked with *ephrin-B1* heterozygous female mice, polydactyl mutants with abnormally developed frontal bones in the skull (called the calvarial phenotype, after the name of the bones). They show that Eph/ephrin signaling regulates gap junction communication, which in turn controls cell sorting. Their results indicate that flawed cell sorting, resulting from dysregulated communication at gap junctions—intracellular membrane channels with pores that allow coupled cells to exchange small molecules—underlies the skeletal abnormalities observed in the mice.

**Figure:**
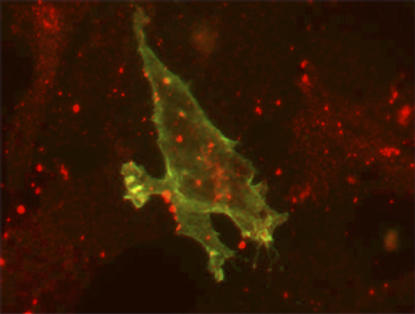
Localization of ephrin-B1 (green) and connexin43 (red) in 3T3 cells.

Previous studies established that *ephrin-B1* heterozygous females exhibit polydactyly while males lacking their copy of *ephrin-B1* and females lacking both copies do not. Polydactyly accompanied a random inactivation of X chromosomes in female cells (in which one X chromosome is silenced in some cells and the second is silenced in others) that created a mosaic pattern of *ephrin-B1* expression, with *ephrin-B1*-expressing cells segregated from cells that didn’t express the gene. Ephrin-B1 mutants also develop multiple defects in tissue derived from neural crest cells—which give rise to cartilage, bone, connective tissue, and other specialized tissues.

In this study, Davy et al. show that the mosaic loss of *ephrin-B1* blocked the differentiation of neural crest cells by disrupting the distribution of a connexin (Cx43) that regulates bone cell differentiation and forms gap junctional pores. Cx43 aggregated between wild-type (nonmutant) cells and between cells that lack *ephrin-B1*, but was rarely seen at the border between *ephrin-B1*-positive and -negative cells, suggesting that the mosaic cells restricted the number of junctional pores. Expression of the ephrin-B1 receptor, EphB2, is elevated in *ephrin-B1*-negative regions in *ephrin-B1* heterozygous embryos, so the researchers suspected that interactions between the receptor and ligand reduced Cx43 levels and disrupted gap junction formation—which they confirmed by tracking gap junction communication in cell cultures. This defect might be mediated by a physical interaction between ephrin-B1 and Cx43.

The researchers propose that gap junction communication is inhibited by interactions between Eph-positive and ephrin-positive cells that cause Cx43 to be sequestered inside cells, where they can’t form gap junction pores and establish cell-to-cell communication—leading to skeletal abnormalities. This explains why the CFNS phenotype is more prevalent in females (who exhibit mosaic expression of *ephrin-B1* through X inactivation). By contributing a mouse model with skull and digit defects that mimic those seen in humans, the researchers have provided a valuable platform for future investigations into the role of ephrins and gap junction communication in disfiguring skeletal disorders.

